# Species diversity and chemical properties of litter influence non-additive effects of litter mixtures on soil carbon and nitrogen cycling

**DOI:** 10.1371/journal.pone.0180422

**Published:** 2017-07-07

**Authors:** Bing Mao, Rong Mao, De-Hui Zeng

**Affiliations:** 1 Key Laboratory of Forest Ecology and Management, Institute of Applied Ecology, Chinese Academy of Sciences, Shenyang, China; 2 Daqinggou Ecological Station, Institute of Applied Ecology, Chinese Academy of Sciences, Shenyang, China; 3 Chongqing Institute of Green and Intelligent Technology, Chinese Academy of Sciences, Chongqing, China; University of Copenhagen, DENMARK

## Abstract

Decomposition of litter mixtures generally cannot be predicted from the component species incubated in isolation. Therefore, such non-additive effects of litter mixing on soil C and N dynamics remain poorly understood in terrestrial ecosystems. In this study, litters of Mongolian pine and three dominant understory species and soil were collected from a Mongolian pine plantation in Northeast China. In order to examine the effects of mixed-species litter on soil microbial biomass N, soil net N mineralization and soil respiration, four single litter species and their mixtures consisting of all possible 2-, 3- and 4-species combinations were added to soils, respectively. In most instances, species mixing produced synergistic non-additive effects on soil microbial biomass N and soil respiration, but antagonistic non-additive effects on net N mineralization. Species composition rather than species richness explained the non-additive effects of species mixing on soil microbial biomass N and net N mineralization, due to the interspecific differences in litter chemical composition. Both litter species composition and richness explained non-additive soil respiration responses to mixed-species litter, while litter chemical diversity and chemical composition did not. Our study indicated that litter mixtures promoted soil microbial biomass N and soil respiration, and inhibited net N mineralization. Soil N related processes rather than soil respiration were partly explained by litter chemical composition and chemical diversity, highlighting the importance of functional diversity of litter on soil N cycling.

## Introduction

Litter decomposition is an important process regulating greenhouse gas emission, soil organic matter formation and nutrient availability for soil biota and plants, and thus is an essential component of C and nutrient cycling in soils in most ecosystems [1, 2]. In both natural and managed ecosystems, because of the species diversity, plant litters with different initial chemical composition generally become mixed and influence the degradation process of organic matter in the soil [3, 4]. Generally, litter decomposition and nutrient release are controlled by chemical traits of substrate and litter mixing effect in terrestrial ecosystems. Examining the effects of litter mixtures’ chemical traits and litter species interactions on soil C and N cycling is therefore of considerable importance in understanding mechanisms of plant-soil interactions.

Many studies have explored changes in soil C and N cycling following litter addition [5–7], and have shown that litter addition has variable (positive or negative) effects [8–11]. However, these studies only examined the effect of litter addition on soil C and N cycling, and failed to consider the effects of litter species diversity. Previous studies have shown that decomposition dynamics of litter mixtures often deviated from the expected values calculated from the average of their component species decomposing alone (i.e., plant litter mixtures generate “non-additive” effects on decomposition) [12]. Mechanisms responsible for non-additive effects of litter mixtures on decomposition include changes in nutrient release and the degradation of organic compounds (e.g. polyphenol, tannin, lignin, cellulose) from different species in litter mixtures. These changes may inhibit or stimulate microbial growth or activity during decomposition of mixed-species litter, and then influence soil C and N cycling [1]. However, most previous studies investigating non-additive effects of litter mixing focused on litter decomposition, but ignored the effects of species mixing on soil C and N cycling [13–15].

Recently, there have been many investigations into the relationship between plant species diversity and ecosystem function [16]. Species diversity includes the numbers of species present (species richness) and the particular species identity (species composition) [17]. Previous studies showed that species interactions can significantly affect litter decomposition, and species diversity might mediate the interaction effects [12, 18, 19]. Regarding the effects of species diversity on soil C and N dynamics, although studies have recently investigated the effects of litter species richness on soil biological processes [14, 20–22], species interaction on soil C and N dynamics and links between plant species composition and soil C and N dynamics remain poorly understood [13, 23].

It is clear that litter chemical traits (e.g. lignin, tannin, cellulose) significantly influence soil C and N cycling in many terrestrial ecosystems [1]. For instance, polyphenols, which are commonly viewed as a group of secondary metabolites in plants, can strongly inhibit soil N cycling [24–26]. Lignin can suppress the activity of decomposer organisms, and therefore limits soil N availability [27, 28]. Moreover, cellulose can be bound by lignin in soil to form stable compounds, which could protect the cell wall compounds from microbial attack, affecting microbial activity [28, 29] and thus soil N availability. These effects of litter chemical traits on soil C and N cycling are mainly concluded from decomposition experiments of individual-species litter, but do not address the effects of litter chemical traits from multiple-species litter decomposition on soil C and N cycling. Regarding the decomposition of mixed-species litter, studies have investigated the contribution of litter chemical traits to the non-additive effects on litter decomposition and soil C and N cycling, but the results are inconsistent [14, 30–32], because the interactions among chemical traits in mixed-species litter decomposition may intricately influence soil C and N cycling [1]. Recently, plant functional traits and their functional diversity have become a significant research interest, providing readers with a broad range of information on understanding the relationships of biodiversity and ecosystem function [33, 34]. An increasing number of studies demonstrate that the effects of species diversity on aboveground processes can be mechanistically understood in terms of chemical properties (chemistry composition and chemical diversity) [31, 35–37]. Moreover, Meier and Bowman [20] found that litter chemical properties could be applied to understanding effects of species diversity on belowground C and N cycling in an alpine moist meadow using a 6-week incubation experiment. How litter chemical properties in mixed-species litter decomposition affect soil C and N cycling during longer time of incubation remains largely unknown and more evidence is needed to support the hypothesis that litter chemical properties rather than litter species richness are potentially important factors affecting soil C and N cycling from other ecosystems amended by mixed-species litter.

In this study, Mongolian pine (*Pinus sylvestris* var. *mongolica*) litter and the litter of three dominant understory species *Artemisia scoparia*, *Setaria viridis* and *Phragmites communis* were chosen to test the effects of species mixing on soil C and N cycling. In a previous study [38], we found that species mixing produced non-additive effects on mass loss, C/N ratio and lignin decomposition of mixed-species litter, and that species composition rather than species richness explained the non-additive effects of species mixing on mass loss. Considering that the decomposition of litter plays a fundamental role in the soil C and N cycling, we hypothesized that (1) litter mixture would produce non-additive effects on soil C and N cycling; (2) species composition rather than species richness may explain the non-additive effects of species mixing on soil C and N cycling, because of differential chemical composition and diversity of component species in litter mixtures. We used soil respiration, net N mineralization, and microbial biomass N measurements to describe soil C and N cycling.

## Materials and methods

### Study site

The plant litter and soil used in the experiment were collected from a 12-year-old Mongolian pine plantation at Daqinggou Ecological Station, Northeast China (42°54′N, 122°21′E; 260 m above sea level). The study site has a dry semiarid climate with a mean annual temperature of 6.4°C. The soil at the study site is classified as a sandy soil (Typic Ustipsamment), with poor soil nutrients (3.15 g kg^-1^ of organic C, 0.24 g kg^-1^ of total N, and 0.09 g kg^-1^ of total P). Dominant understory species in the Mongolian pine plantation include *Artemisia scoparia*, *Setaria viridis*, *Phragmites communis*, and *Leonurus sibiricus* [39].

### Litter and soil incubation experiment

Mongolian pine and three understory species (*A*. *scoparia*, *S*. *viridis* and *P*. *communis*) were used in our incubation experiment. Leaf litter of Mongolian pine and aboveground residue of *A*. *scoparia*, *S*. *viridis* and *P*. *communis* were collected from the 12-year-old Mongolian pine plantation in October 2011. Litter was cut into pieces of 1 cm long in order to reduce the influence of litter size on litter decomposition, and then the litter was stored in paper bags at room temperature until experimental use. Soil at 0–10 cm layer was also collected from the 12-year-old Mongolian pine plantation because this is the most active part of the soil. The soils were mixed thoroughly after roots and organic residues were removed from the soils. After sieving (2 mm), the soils were divided into two sub-samples. One was used for analyses of initial NH_4_^+^-N and NO_3_^-^-N concentrations and microbial biomass N. The other was stored at 4°C and used for the incubation.

Four single litter species and their mixtures comprised of all 11 possible 2-, 3- and 4-species combinations of equal mass proportion were incubated with 80 g (dry weight) soil in plastic cups (polyvinyl chloride) [38]. In total, there were 15 treatments and each was 4 replications (blocks) in each incubation stage (14, 42, 84 and 182 days). Thus, there were a total of 240 plastic cups as microcosms (15 treatments × 4 incubation periods × 4 replications) in our incubation experiment. Soil samples (60 cups, 15 treatments ×4 replications) were collected at each incubation time (14, 42, 84 182 days). For each litter treatment, one gram of litter per replicate was placed on the surface of soil. Perforated adhesive films were used to cover plastic cups for the aims of reducing evaporation while allowing gaseous exchange. After 14, 42, 84 and 182 days of incubation (25°C) in an incubator, soil inorganic N (NH_4_^+^-N and NO_3_^-^-N) and soil microbial biomass N were determined. We quantified litter mixing effects on soil C and N cycling by measuring soil basal respiration, net N mineralization rate, and microbial biomass N.

The concentration of soil inorganic N (NH_4_^+^-N and NO_3_^-^-N) was determined by shaking 20-g fresh soil with 50 mL 2 mol L^-1^ KCl solution for 30 min on a reciprocal shaker [40]. The soil solutions were analyzed for NH_4_^+^-N and NO_3_^-^-N concentrations on a continuous flow autoanalyzer (AutoAnalyzer III, Bran+Luebbe GmbH, Germany). Soil net N mineralization rates were determined by the subtraction between the initial and final inorganic N concentrations at each incubation interval (14, 42, 84 and 182 days) [20].

Soil microbial biomass N was measured using the chloroform fumigation-extraction method [41]. Soil samples from each treatment were divided into two sub-samples. One sub-sample was fumigated with alcohol-free chloroform for 24 h in an evacuated desiccator, and the other one was not. Fumigated samples and unfumigated samples were extracted with 50 mL 0.5 mol L^-1^ K_2_SO_4_ and shaken for 1 h on a reciprocating shaker. Extractable total N concentration was analyzed by the alkaline persulfate oxidation method [42].

A beaker containing 10 mL 0.2 mol L^-1^ NaOH was placed in each plastic cup to trap the evolved CO_2_. The cups were incubated in a dark incubator at 25°C. At 4–6 days of interval, the CO_2_ evolution was determined by titration of NaOH solution with 0.1 mol L^-1^ HCl in an excess of BaCl_2_, and phenolphthalein was an indicator. After the NaOH beaker was taken out, the air in the cup was replenished by opening it for about 4 hours. Soil water content was adjusted with distilled water to 60% of water-holding capacity following air sampling. The NaOH beaker was replaced at each sampling.

### Litter chemical analyses

We quantified chemical diversity and chemical composition of litter by measuring initial litter total C and N, and six carbon chemical compounds (lignin, cellulose, soluble sugar, total polyphenol, hydrolyzable polyphenol and condensed tannin). We obtained initial C concentration using the K_2_Cr_2_O_7_–H_2_SO_4_ wet oxidation method of Walkley and Black [43], and total N concentration using a continuous-flow autoanalyzer (AutoAnalyzer III, Bran+Luebbe GmbH, Germany). A modified acetyl bromide method and an acid-hydrolysis method were used to assess the concentrations of litter lignin and cellulose, respectively [44, 45]. Soluble sugar concentration of litter sample was determined using anthrone method [46]. Concentrations of total and hydrolyzable polyphenols were determined by the Folin-Ciocalteu method [47]. Condensed tannin was measured according to the acid butanol method [48].

### Data analyses

General linear model (GLM) of repeated measures (SPSS 16.0), using Type I sums of squares (SS), was performed to test for additive or non-additive effects of species mixing on soil C and N cycling. Following the methodology of Ball et al. [49], a significant SpInt (species interaction) term (and/or its interaction with time) indicates a significant non-additive effect of multiple-species mixtures (see S1 Table). This term had 11 levels, each representing one of the multiple-species combinations. Then we replaced the significant SpInt term with a Richness term (the number of species present in species combinations, 1, 2, 3, or 4) and Composition term (15 possible combinations in monocultures and mixtures) using the model of repeated measures to evaluate if the non-additive effects were mediated by richness and/or composition. In the model, incubation time was treated as the within-subject effect, previously log-transformed to meet normality.

Paired *t*-test was used to evaluate the direction (synergistic or antagonistic) of non-additive effect by determining differences between observed and expected values [50, 51]. According to Gartner and Cardon [12], species-mixing effects of litter on soil C and N cycling were classified as follows: additive effects (no significant differences between observed and expected values), synergistic non-additive effects (observed values were significantly higher than expected values), and antagonistic non-additive effects (observed values were significantly lower than expected values).

To test whether non-additive soil responses to litter mixtures were influenced by litter chemical diversity (*H*_c_) and chemical composition, simple linear regressions were used. Meanwhile, linear regressions between the relative abundance of each understory species in the litter mixtures and soil responses were used to detect the effects of progressive loss of these species (decreases in relative abundance) on non-additive soil C and N responses to litter mixtures. The relative abundance of a given species ranged between 0 and 50% in mixtures. Significance was evaluated at *α* = 0.05 in all cases. The concentration of each chemical trait within a given litter mixture was calculated by averaging values of each chemical trait from the component species in the mixture.

For all multiple-species mixtures, the expected soil responses (*E*) were calculated according to Eq (1):

$$\mathrm{Expected}\;\mathrm{value}\;(E)=\left(\sum_{i=1}^{S}R_{i}\right)/S$$ (1)

where *R*_*i*_ is the observed soil response when species *i* was added alone (raw data was shown in S1 Fig), and *S* is the total number of species in the litter mixture (results was shown in S2 Fig, S3 Fig and S4 Fig). For calculation of observed values (*O*) of net N mineralization, total inorganic N (TIN) at the end of 14, 42, 84 and 182 days of incubation period was subtracted from TIN values at the beginning of each incubation interval. In our study, negative (*O*–*E*)/*E* values for the net N mineralization response mean the *O* value was more negative than expected (*E*) value, which indicated that there was more net N immobilization than expected [13].

To describe the chemical composition of the litter mixtures, we analyzed the initial chemical traits (day 0) of the four individual species that were used to construct the litter mixtures with a principal component analysis (PCA) (Canoco 5.0). Initial litter chemical traits of four species were standardized (using the “standardized species” option) and were log-transformed before conducting unconstrained PCA. Then, we used PC scores from the four single species rather than concentrations of the chemical traits themselves to calculate the chemical composition of litter mixtures because some chemical traits co-varied with each other and were not statistically independent [20, 13]. We calculated PC scores for each litter mixture by averaging the PC scores associated with each species in the mixture according to Eq (2):

$$PCX=\left(\sum_{i=1}^{S}PCX_{i}\right)/S$$ (2)

where *PCX* is the PC score for axis *X* for each litter mixture (where *X* is either 1, 2, or 3), *PCX*_*i*_ is the PC score for axis *X* for species *i*, and *S* is the total number of species in the mixture. Non-additive soil responses to the litter mixtures were then statistically modeled as a function of the litter mixture PC scores according to Eq (3):

R=f(PC1×PC2×PC3) (3)

where *R* (either soil respiration, soil net N mineralization, or soil microbial biomass N) is the non-additive soil response for the mixtures, and *f* is a multiple regression model. For all regression analyses, we used log(*x* + 1) transformed (*O*–*E*)/*E* values for soil respiration, net N mineralization and microbial biomass N, where *O* and *E* represent observed and expected values, respectively. The expected soil responses (*E*) were calculated according to Eq (1). Data was examined for homogeneity of variance with fitted versus residual plots, and for normality of residuals with quantile-quantile plots.

Chemical diversity (*H*_c_) of each litter mixture was calculated from the initial concentration of each litter mixture using the Shannon diversity index according to Eq (4):

$$H_{c}=\text{-}\sum_{i=1}^{n}p_{i}\ln p_{i}$$ (4)

where *n* is the total number of chemical traits present in a given litter treatment, and *p*_*i*_ is the mass proportion of chemical property *i* in the litter mixture (results was shown in S1 Table).

## Results

### Initial litter chemical traits of four species used in the incubation

*A*. *scoparia* had significantly higher litter N concentration than the other three species, and there was no significant difference of N concentration among Mongolian pine, *S*. *viridis* and *P*. *communis* (Table 1). Pine generally had higher lignin, soluble sugar, polyphenol, condensed tannin, and hydrolysable polyphenol than the other three species. There was no significant difference of cellulose concentration among the four species.

**Table 1:** Initial litter chemical traits (±SE, *n* = 4) of four species.

Chemical traits	Mongolian pine	*A*. *scoparia*	*S*. *viridis*	*P*. *communis*
N (mg g^-1^)	3.6 (0.93)^b^	13.9(2.11)^a^	3.8(0.26)^b^	4.5 (0.86)^b^
C/N	152.7(2.5)^a^	35.3(6.3)^c^	115.5(7.3)^b^	104.9(1.3)^b^
Lignin (mg g^-1^)	377.4(21.1)^a^	345.7(14.2)^b^	342.6(13.2)^b^	357.7(21.4)^ab^
LG/N	101.0(4.1)^a^	23.3(0.7)^d^	90.6(5.5)^b^	73.9(3.8)c
Cellulose (mg g^-1^)	114.5(6.4)^a^	114.3(9.7)^a^	121.0(6.8)^a^	112.6(8.4)^a^
(LG+CL)/N	136.5(10.2)^a^	30.9(1.0)^c^	122.7(7.8)^a^	96.5(5.2)^b^
Soluble sugar (mg g^-1^)	58.5(3.8)^a^	10.9(0.7)^d^	32.5 (2.2)^b^	23.0(2.1)^c^
Polyphenol (mg g^-1^)	14.9 (1.6)^a^	9.9(0.2)^b^	5.0(0.1)^c^	5.2(0.5)^c^
(LG+PL)/N	108.8(7.8)^a^	24.2(0.7)^d^	91.9(5.6)^b^	74.9(3.8)^c^
Condensed tannin (mg g^-1^)	7.3 (0.5)^a^	1.7 (0.03)^b^	0.8 (0.03)^c^	1.6 (0.13)^b^
Hydrolyzable polyphenol (mg g^-1^)	11.5(0.1)^a^	6.4(0.3)^b^	4.1(0.1)^c^	4.0(0.1)^d^

Different letters (a, b, c and d) in rows indicate statistical difference among different species according to Tukey’s test (*P* < 0.05). LG/N: lignin/N; (LG+CL)/N: (lignin+cellulose)/N; (LG+PL)/N: (lignin+polyphenol)/N

**Fig 1:**
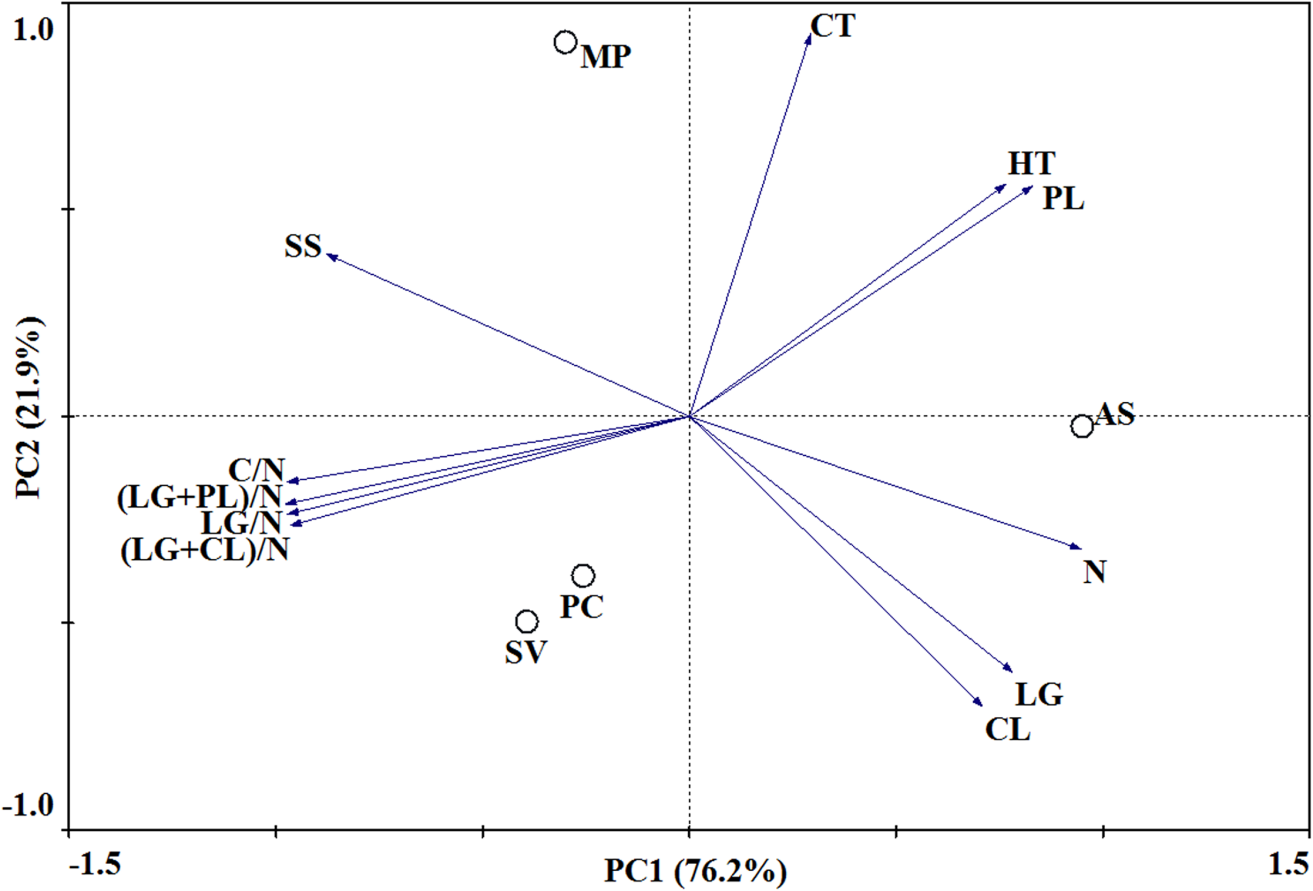
Principal component plot of 11 litter chemical traits of four litter species. The first two principal components (PCs) accounted for 76.2% (PC1) and 21.9% (PC2) of the total variance, respectively. LG: lignin; CL: cellulose; SS: soluble sugar; PL: polyphenols; HP: hydrolyzable polyphenol; CT: condensed tannin; LG/N: lignin/N ratio; (LG+PL)/N: ratio of (lignin + polyphenol)/N; (LG+CL)/N: ratio of (lignin + cellulose)/N; MP: Mongolian pine; AS: *A*. *scoparia*; SV: *S*. *viridis*; PC: *P*. *communis*.

Principal component analyses (PCA) showed that the Mongolian pine and *S*. *viridis* were clearly separated, while *S*. *viridis* and *P*. *communis* were not (Fig 1). Ratios of C/N, lignin/N, (lignin+cellulose)/N and (lignin+polyphenol)/N and concentrations of N and polypehnol were separated on the first axis, and concentrations of lignin, cellulose and condensed tannin were separated on the second axis. Here, PC1and PC2 altogether explained 98.1% of the variation in the chemical traits.

### Non-additive soil C and N responses to mixed-species litter

**Fig 2:**
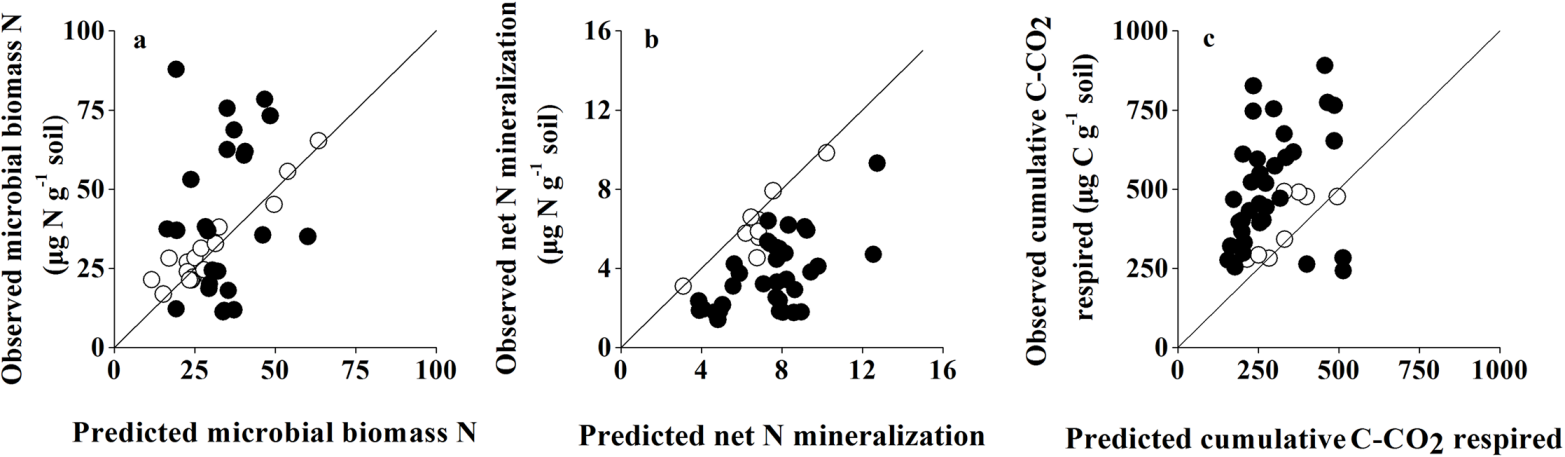
**Observed soil microbial biomass N (a), soil net N mineralization (b) and cumulative soil respiration (c) in relation to the expected values calculated from the corresponding monoculture treatments.** The line indicates the 1:1 relationship along which observed and expected values are equal. Data points represent averages across treatments over time. ●: non-additive effects, ○: additive effects.

**Fig 3:**
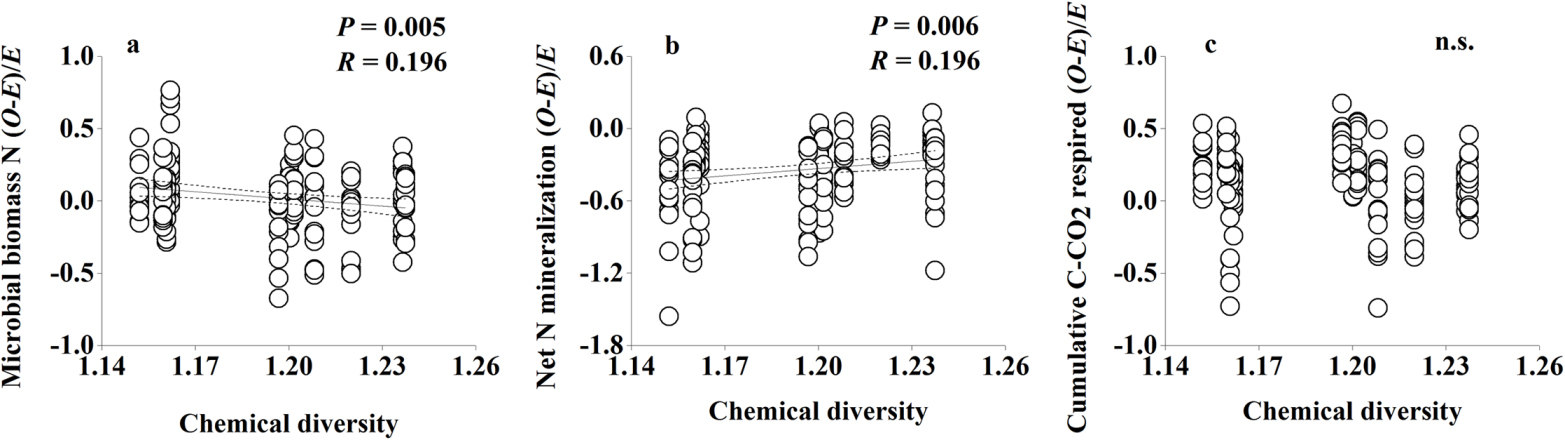
**Non-additive soil microbial biomass N (a), soil net N mineralization (b) and cumulative soil respiration (c) responses to litter mixtures as a function of chemical diversity (*H*_c_) of litter mixtures.** Dashed lines represent the 95% confidence interval of the regression. *O*: observed values; *E*: expected values. n.s.: not significant.

**Fig 4:**
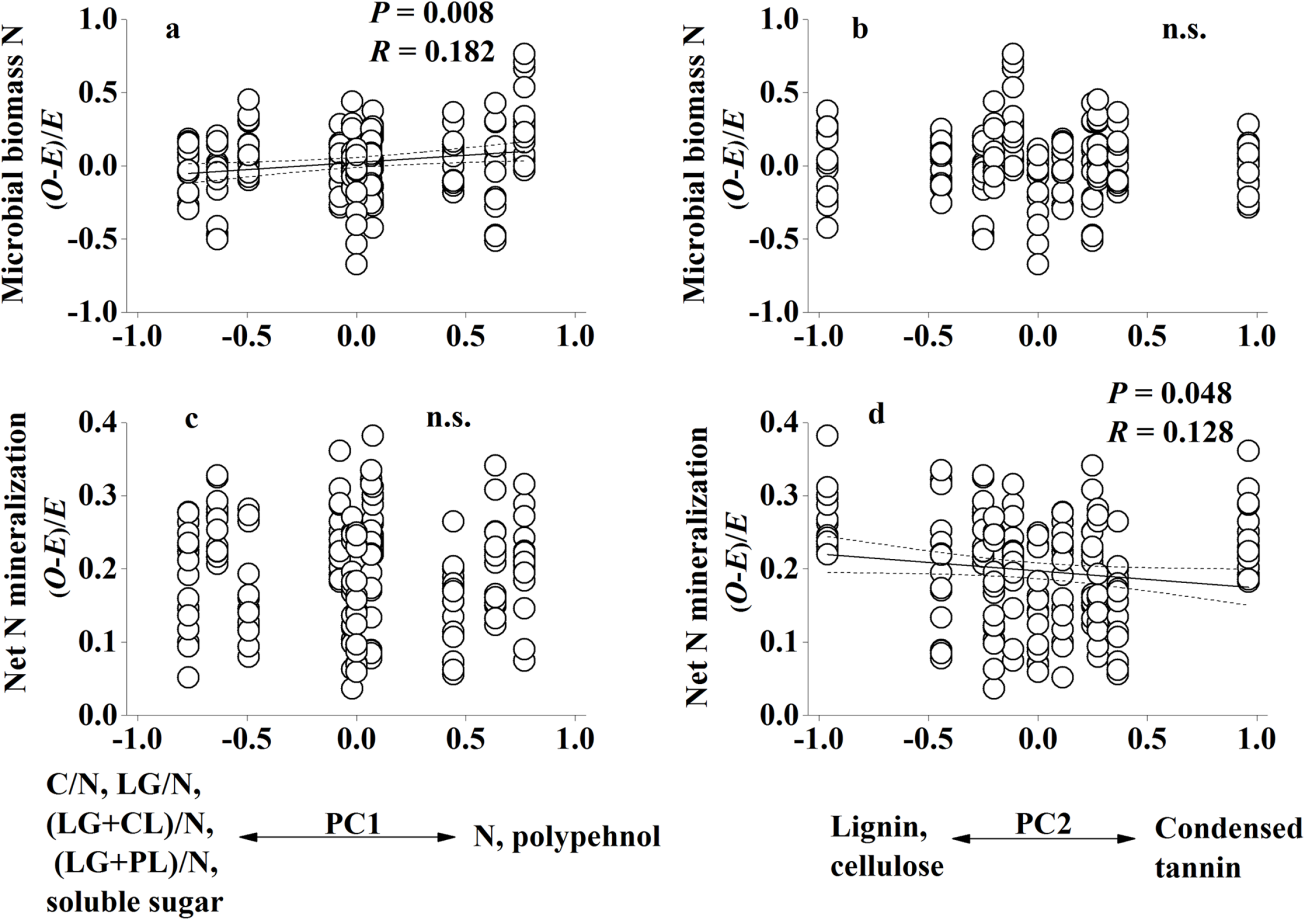
**Non-additive soil microbial biomass N (a and b) and net N mineralization (c and d) response to litter mixtures as a function of the first and second PC axes, respectively, that were used to describe chemical composition of litter.** Dashed lines represent the 95% confidence interval of the regression. *O*: observed values; *E*: expected values. n.s.: not significant.

Non-additive effects of species mixing were recorded for soil microbial biomass N, net N mineralization and cumulative soil respiration (Day×SpInt term all at *P*<0.001; Table 2). Non-additive microbial biomass N responses to litter mixtures were found in 54.5% of cases for all tested mixtures (Table 3; Fig 2A); synergistic non-additive effects (observed values was higher than expected values) were slightly more common than antagonistic non-additive effects (observed values was lower than expected values), with 13 vs 11 cases (Table 3; Fig 2A). Replacing the SpInt term with Richness and Composition identified that non-additive soil microbial biomass N responses to mixed-species litter were not significantly regulated by species richness (Day×Richness term at *P* = 0.412; Table 4), but significantly regulated by species composition (Day×Composition term at *P* = 0.036; Table 4). Linear regression analyses revealed that non-additive soil microbial biomass N responses to mixed-species litter had significant relationships with litter chemical diversity (*H*_c_) (*P* = 0.005; Fig 3A). Non-additive soil microbial biomass N responses to litter mixtures had a significant relationship with PC1 scores (*P* = 0.008; Fig 4A).

**Table 2:** Summary of the ANOVA’s test for additive and non-additive effects of species mixing on soil microbial biomass N, soil net N mineralization and soil respiration using Type I sums of squares (SS).

Soil responses to litter mixtures		*SS*	*df*	*F*	*P*
Microbial biomass N	Block	0.04	3	2.59	0.071
	Mongolian pine	0.00	1	0.14	0.711
	*A*. *scoparia*	0.28	1	50.71	<0.001
	*S*. *viridis*	0.00	1	0.57	0.458
	*P*. *communis*	0.11	1	20.54	<0.001
	SpInt	0.81	6	24.77	<0.001
	Error	0.16	30		
	Day	0.09	1	15.94	<0.001
	Day × Block	0.01	3	0.59	0.629
	Day × Mongolian pine	0.00	1	0.10	0.759
	Day × *A*. *scoparia*	0.48	1	82.34	<0.001
	Day × *S*. *viridis*	0.42	1	71.56	<0.001
	Day × *P*. *communis*	0.32	1	54.84	<0.001
	Day × SpInt	0.55	6	15.76	<0.001
	Error	0.17	30		
N mineralization	Block	0.02	3	0.37	0.779
	Mongolian pine	0.03	1	1.57	0.220
	*A*. *scoparia*	0.23	1	13.06	0.001
	*S*. *viridis*	1.92	1	111.50	<0.001
	*P*. *communis*	0.16	1	9.55	0.004
	SpInt	0.42	6	4.05	0.004
	Error	0.52	30		
	Day	0.46	1	23.86	<0.001
	Day × Block	0.07	3	1.12	0.357
	Day × Mongolian pine	0.02	1	1.05	0.314
	Day × *A*. *scoparia*	0.00	1	0.11	0.745
	Day × *S*. *viridis*	0.42	1	21.69	<0.001
	Day × *P*. *communis*	0.00	1	0.08	0.779
	Day × SpInt	0.87	6	7.53	<0.001
	Error	0.58	30		
Cumulative C-CO_2_ respired	Block	0.01	3	0.77	0.519
	Mongolian pine	0.07	1	20.68	<0.001
	*A*. *scoparia*	0.08	1	24.91	<0.001
	*S*. *viridis*	1.24	1	388.44	<0.001
	*P*. *communis*	0.91	1	286.89	<0.001
	SpInt	0.39	6	20.16	<0.001
	Error	0.10	30		
	Day	0.17	1	42.99	<0.001
	Day × Block	0.02	3	1.56	0.221
	Day × Mongolian pine	0.01	1	1.77	0.194
	Day × *A*. *scoparia*	0.25	1	61.90	<0.001
	Day × *S*. *viridis*	0.02	1	5.85	0.022
	Day × *P*. *communis*	0.00	1	0.66	0.422
	Day × SpInt	0.15	6	6.16	<0.001
	Error	0.12	30		

SpInt is abbreviated as species interaction. Day means incubation days

**Table 3:** The number and percentage of additive effects and non-additive effects of species mixing on soil microbial biomass N, soil N mineralization and soil respiration.

Soil responses to litter mixtures		Additive effects	Non-additive effects		
			Total	Synergistic effects	Antagonistic effects
Microbial biomass N	Number	20	24	13	11
	Percentage (%)	45.5	54.5	38.2	32.4
N mineralization	Number	10	34	0	34
	Percentage (%)	22.7	77.3	0.0	100.0
Cumulative C-CO_2_ respired	Number	10	34	31	3
	Percentage (%)	22.7	77.3	91.2	8.8

**Table 4:** Summary of the ANOVA’s test to evaluate if richness and/or composition influence the non-additive effects of species mixing.

Soil responses to litter mixtures		*SS*	*df*	*F*	*P*
Microbial biomass N	Richness	79.01	3	149.02	<0.001
	Composition	44.98	11	23.14	<0.001
	Error	7.95	45		
	Day	0.01	1	0.11	0.745
	Day × Richness	0.32	3	0.98	0.412
	Day × Composition	2.54	11	2.14	0.036
	Error	4.85	45		
N mineralization	Richness	2.89	3	19.60	<0.001
	Composition	1.36	11	2.52	0.014
	Error	2.21	45		
	Day	0.43	1	5.63	0.022
	Day × Richness	0.18	3	0.78	0.510
	Day × Composition	2.02	11	2.40	0.019
	Error	3.44	45		
Cumulative C-CO_2_ respired	Richness	3.24	3	213.48	<0.001
	Composition	1.66	11	29.86	<0.001
	Error	0.23	45		
	Day	0.60	1	78.72	<0.001
	Day × Richness	0.24	3	10.58	<0.001
	Day × Composition	0.45	11	5.34	<0.001
	Error	0.34	45		

Day means incubation days

Non-additive effects of species mixing were recorded for soil net N mineralization (Day×SpInt term at *P*<0.001; Table 2). Non-additive soil net N mineralization responses were found in 77.3% of cases for all tested mixtures (Table 3; Fig 2B). Soil net N mineralization was always lower than expected values in litter mixtures (Table 3; Fig 2B). Replacing the SpInt term with Richness and Composition identified that non-additive soil net N mineralization responses to mixed-species litter were not significantly regulated by species richness (Day×Richness term at *P* = 0.510; Table 4), but significantly regulated by species composition (Day×Composition term at *P* = 0.019; Table 4). Linear regression analyses revealed that non-additive soil net N mineralization responses to mixed-species litter had significant relationships with litter chemical diversity (*H*_c_) (*P* = 0.006; Fig 3B). Meanwhile, non-additive soil net N mineralization responses to mixed-species litter showed a significant correlation with PC2 scores (*P* = 0.048; Fig 4D).

Non-additive effects of species mixing were recorded for soil respiration (Day×SpInt term at *P*<0.001; Table 2). Non-additive soil respiration responses were found in 77.3% of cases for all tested mixtures (Table 3; Fig 2C). Cumulative soil respiration showed more frequent synergistic effects than antagonistic effects, with 31 and 3 cases respectively (Table 3; Fig 2C). Replacing the SpInt term with Richness and Composition identified that non-additive soil respiration responses to mixed-species litter were significantly regulated by both species richness and species composition (Day×Richness term and Day×Composition term all at *P*<0.001; Table 4). Non-additive soil respiration responses to mixed-species litter showed no significant correlation with litter chemical diversity (*H*_c_) (*P>*0.05; Fig 3C). There was no significant relationship between PCA scores of litter chemical traits and non-additive soil respiration responses to mixed-species litter.

### Effects of understory species loss on non-additive soil C and N responses to mixed-species litter

**Fig 5:**
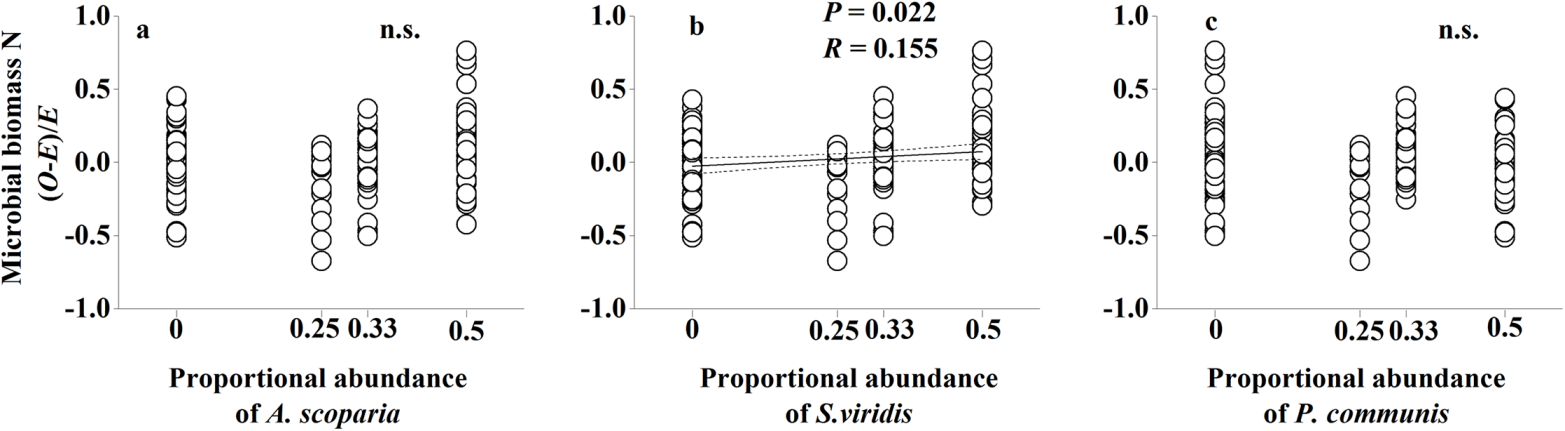
Non-additive soil microbial biomass N responses to mixed-species litter as a function of individual species abundance of *A*. *scoparia*, *S*. *viridis* and *P*. *communis* within the litter mixtures. Dashed lines represent the 95% confidence interval of the regression. *O*: observed values; *E*: expected values. n.s.: not significant.

**Fig 6:**
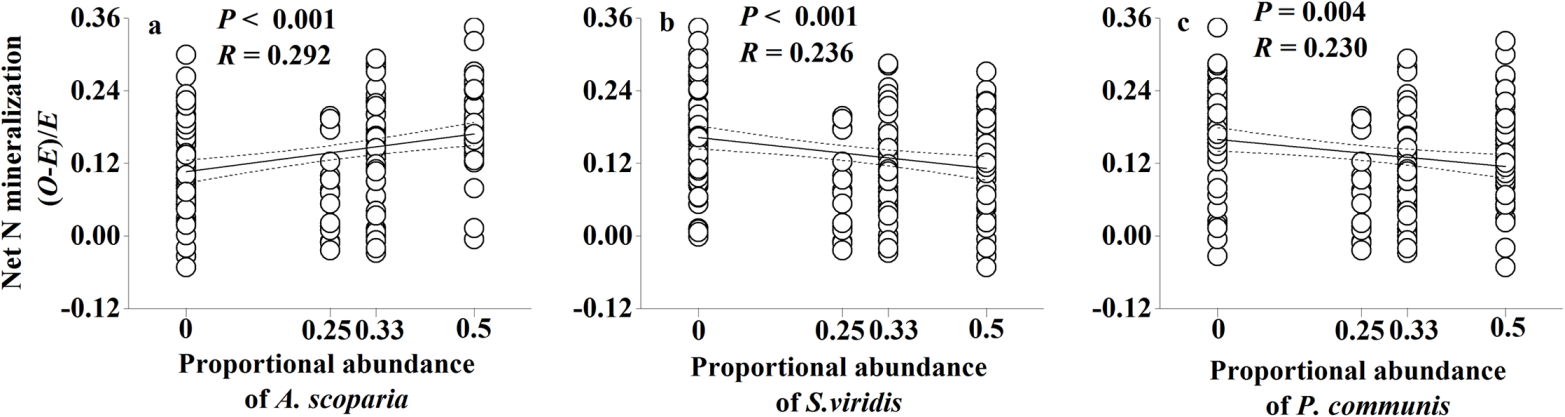
Non-additive soil net N mineralization responses to mixed-species litter as a function of individual species abundance of *A*. *scoparia*, *S*. *viridis* and *P*. *communis* within the litter mixtures. Dashed lines represent the 95% confidence interval of the regression. *O*: observed values; *E*: expected values. n.s.: not significant.

**Fig 7:**
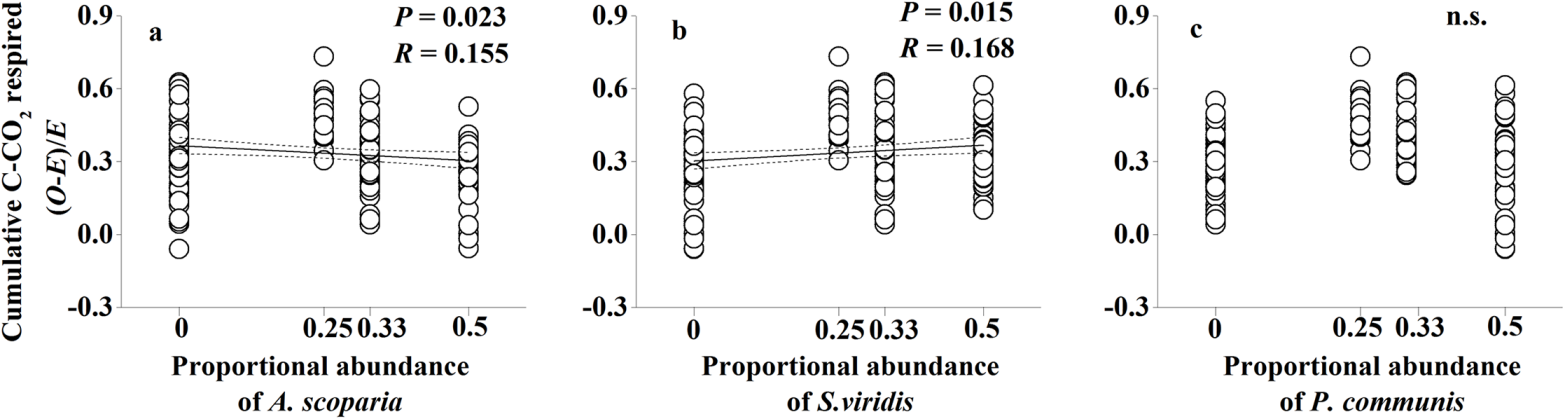
Non-additive soil respiration responses to mixed-species litter as a function of individual species abundance of *A*. *scoparia*, *S*. *viridis* and *P*. *communis* within litter the mixtures. Dashed lines represent the 95% confidence interval of the regression. *O*: observed values; *E*: expected values. n.s.: not significant.

To detect which individual understory species contributed to non-additive effects, we compared the proportional abundance of individual species within litter mixtures against non-additive soil C and N responses to litter mixtures. We found that the abundance of *S*. *viridis* litter showed a significant positive correlation with non-additive microbial biomass N responses to mixed-species litter (*P* = 0.022; Fig 5B). The abundance of each of three understory species showed a significant effect on non-additive net N mineralization responses to mixed-species litter (*P*<0.001, *P*<0.001, *P* = 0.004, respectively; Fig 6). Meanwhile, the abundance of *A*. *scoparia* litter showed a significant negative effect and the abundance of *S*. *viridis* litter showed a significant positive effect on non-additive cumulative soil respiration (*P* = 0.023 and *P* = 0.015, respectively; Fig 7A and 7B).

## Discussion

In this study, non-additive effect on soil C and N cycling was more common than additive effect. Moreover, we observed a prevalence of synergistic non-additive effects on soil respiration and soil microbial N over antagonistic non-additive effects, and a prevalence of antagonistic non-additive effects on soil net N mineralization. Our results were in line with the majority of limited studies regarding the effect of litter mixtures on C and N dynamics in soils [13, 23, 52], further confirming that litter mixture-induced changes in soil C and N cycling could not be predicted by the values derived from single species litters.

We used the Shannon diversity index (*H*_c_) to describe the chemical diversity of our litter mixtures. We found greater differences between observed and expected N immobilization and microbial biomass N as chemical diversity increased, indicating that high chemical dissimilarity of litter species in litter mixtures was conducive to the soil N transformation, similar to Meier and Bowman [13]. Our results present additional evidence to the complementarity hypothesis mentioned by Hättenschwiler et al. [1]: by mixing high- and low-quality litters, easily decomposable resources are available to decomposers, eventually leading to a general high nutrient availability in the mixture and allowing nutrient transfer to the low-quality litter, thus enhancing decomposition of mixed-species litter and soil N cycling. Meanwhile, chemical composition (calculated from PCA) was also used to investigate the contribution of litter chemical traits to the non-additive effects on soil C and N cycling in our study. We found that chemical composition of litter mixtures also showed significant positive correlation with non-additive soil N immobilization and microbial biomass N, indicating that specific compounds in litter mixtures also strongly influenced the non-additive N immobilization and microbial biomass N. Thus, one or several litter chemistry traits (e.g. N, C/N ratio, lignin/N or phenolic/N) might be insufficient to understanding soil N cycling [32].

Previously studies found that species diversity might mediate the interaction effects of species mixing on decomposition of mixed-species litter [12, 18]. We previously found that species composition rather than species richness mediated mass loss of mixed-species litter [38]. Similarly, according to GLM analysis, we found that species composition rather than species richness mediated positive effects of species-mixing on soil N immobilization and microbial biomass N responses to mixed-species litter. The significant regulation of species composition on non-additive soil microbial biomass N responses might be due to the litter chemical composition. For instance, *S*. *viridis* litter with high ratios of C/N, lignin/N, (lignin+cellulose)/N, and (lignin+polyphenol)/N in litter mixture might inhibit N utilization by soil microbes. Thus, litter chemical diversity and chemical composition, but not species richness, contributed to non-additive effects of mixed-species litter on soil microbial biomass N in our study, similar to Jiang et al. [14], Bottollier-Curtet et al. [53], and Chen et al. [22].

In general, litter input is expected to increase C mineralization in soil [54–56]. Similarly, we found that mixed-species litter significantly promoted soil respiration. Previous studies suggested that differences in initial litter chemical traits combined with increasing litter inputs could result in potential changes in soil respiration [1, 57]. In a previous study [38], we found that mass loss of litter mixtures was slightly more often promoted by species mixing. Regarding litter chemical properties (chemical diversity and chemical composition), we found that non-additive soil respiration amended by mixed-species litter was not significantly regulated by chemical diversity and chemical composition in this study, different from Meier and Bowman [20], who found that soil respiration tended to be changed when chemically distinct litter species were assembled. Moreover, according to GLM analysis, we found that species composition and richness mediated species-mixing effects on soil respiration responses to mixed-species litter. Difference of physical characteristics of leaf litter, such as leaf litter surface area and shape, could potentially be a major reason for the non-additive soil respiration responses to mixed-species litter in this study. For instance, *S*. *viridis* litter had greater surface area than *A*. *scoparia* litter, indicating that *S*. *viridis* litter had higher water retention capacity than *A*. *scoparia* litter. Higher water retention capacity by some litter types could be beneficial to the microbial activity, and thus beneficial to the soil C mineralization amended by the litter mixtures of contrasting morphology [58, 59]. Meanwhile, soil biota might be another reason for the non-additive soil respiration responses to mixed-species litter, because of differences in the attractiveness of certain litter types to different species of invertebrates and microhabitat diversity [1, 59, 60].

We found that loss of the three individual understory species from litter mixtures differentially affected non-additive responses of soil microbial biomass N, N mineralization and respiration, with removal of *S*. *viridis* significantly affecting the responses of soil microbial N and respiration, and removal of *A*. *scoparia* and *P*. *communis* significantly affecting the responses of soil N mineralization. However, in terms of correlation coefficients (*R*), the loss of individual understory species from litter mixtures partially account for the non-additive effects of species mixing on soil C and N cycling. Indeed, there are many other factors affecting soil C and N cycling, such as litter chemical traits and soil microbial activity [1]. During litter decomposition, soil microbial community and microbial activity could be significantly affected by the decomposition of labile organic matter (e.g. tannin, polyphenol) and recalcitrant organic matter (e.g. lignin, cellulose) of litter mixtures, and thus resulting in antagonistic or synergistic non-additive effects of species mixing on soil C and N cycling [28, 29]. Regretfully, we did not examine any parameters of microbial activity and microbial community, so the inference based on previous literature needs to be verified in future studies.

Understory plant community can increase forest nutrient retention, and aboveground litter addition can stimulate soil biogeochemical processes (e.g. microbial biomass, soil respiration) and increased soil C storage [61, 62]. Across temperate zone forests, understory herbs only make up an average of 0.2% of aboveground biomass, but provide approximately 16% of annual litterfall, and herbaceous litter decomposes more than twice as rapidly as tree litter [63]. The soils in Mongolian pine plantation ecosystem are notoriously deficient in N and P nutrients, and pine litter showed much slower decomposition rate than the understory species litter [39]. Thus, loss of understory species, due to grazing and litter raking, could exacerbate the decline of the Mongolian pine plantation. Previously, we found that the addition of the three understory species litter could promote pine litter decomposition in litter mixtures, which is conducive to the nutrient release from litter decomposition to soil [38]. Furthermore, this study found that the addition of the three understory species litter in mixed-species litter showed significant effects on soil N mineralization, and *S*. *viridis* showed significant influence on soil microbial biomass N and respiration. Thus, understory species should be appropriately maintained in the Mongolian pine plantation. Regretfully, only three understory species were used in our study. More understory species should be used to test the addition effects of mixed-species on litter decomposition and soil C and N cycling in future studies for the aims to understanding the role of understory species in maintaining the structure and function of the Mongolian pine plantation.

Moreover, according to the information of the effects by progressive understory species loss on non-additive soil responses to mixed-species litter in our study, we might predict the consequence of understory species loss and/or gain on soil C and N cycling amended by litter mixtures in the Mongolian pine plantations, which provides a theoretical basis for the management of litter. For example, reduced abundance of *A*. *scoparia* litter decreased soil net N mineralization and increased soil respiration.

## Conclusions

Mixed-species litter produced a prevalence of synergistic non-additive effects on soil N immobilization, soil microbial biomass N and soil respiration. Chemical properties (chemical diversity and chemical composition) rather than species richness per se regulated the non-additive effects of mixed-species litter on soil N immobilization and microbial biomass N, while the influence of mixed-species litter on soil respiration depended on species diversity (species composition and species richness) rather than litter chemical properties. Additionally, loss of understory species had important effects on non-additive soil N immobilization, soil microbial biomass N and soil respiration amended by mixed-species litter, and different chemical compositions of litter might explain the effects of understory species loss. This study found that litter chemical properties showed more important effects on soil N cycling than species richness in the Mongolian pine plantations, and provided an opportunity to understand the understory species loss on belowground soil ecological processes.

## Supporting information

S1 Table Species diversity (*H*_c_), species richness and species composition of single litter species and their mixtures. (DOCX)

S1 Fig **Soil microbial biomass N (a), soil net N mineralization (b) and soil respiration (c) amended by litter in monoculture at 14, 42, 84 and 182 days of incubation.** Data are means ± SE, with *n* = 4. MP: Mongolian pine; AS: *A*. *scoparia*; SV: *S*. *viridis*; PC: *P*. *communis*.
(DOCX)

S2 Fig Observed and expected soil microbial biomass N amended by litter mixtures at 14, 42, 84 and 182 days of incubation. Data are means ± SE, with *n* = 4. MP+AS: mixture of Mongolian pine + *A*. *scoparia*; MP+SV: mixture of Mongolian pine + *S*. *viridis*; MP+PC: mixture of Mongolian pine + *P*. *communis*; AS+SV: mixture of *A*. *scoparia* + *S*. *viridis*; AS+PC: mixture of *A*. *scoparia* + *P*. *communis*; SV+PC: mixture of *S*. *viridis* + *P*. *communis*; MP+AS+SV: mixture of Mongolian pine, *A*. *scoparia* and *S*. *viridis*; MP+AS+PC: mixture of Mongolian pine, *A*. *scoparia* and *P*. *communis*; MP+SV+PC: mixture of Mongolian pine, *S*. *viridis* and *P*. *communis*; AS+SV+PC: mixture of *A*. *scoparia*, *S*. *viridis* and *P*. *communis*; MP+AS+SV+PC: mixture of Mongolian pine, *A*. *scoparia*, *S*. *viridis* and *P*. *communis*. Expected soil microbial biomass N amended by mixtures was calculated from the values amended by monocultures (S1A Fig) at 14, 42, 84 and 182 days of incubation according to Eq (1).
(DOCX)

S3 Fig Observed and expected soil net N mineralization amended by litter mixtures at 14, 42, 84 and 182 days of incubation. Data are means ± SE, with *n* = 4. MP+AS: mixture of Mongolian pine + *A*. *scoparia*; MP+SV: mixture of Mongolian pine + *S*. *viridis*; MP+PC: mixture of Mongolian pine + *P*. *communis*; AS+SV: mixture of *A*. *scoparia* + *S*. *viridis*; AS+PC: mixture of *A*. *scoparia* + *P*. *communis*; SV+PC: mixture of *S*. *viridis* + *P*. *communis*; MP+AS+SV: mixture of Mongolian pine, *A*. *scoparia* and *S*. *viridis*; MP+AS+PC: mixture of Mongolian pine, *A*. *scoparia* and *P*. *communis*; MP+SV+PC: mixture of Mongolian pine, *S*. *viridis* and *P*. *communis*; AS+SV+PC: mixture of *A*. *scoparia*, *S*. *viridis* and *P*. *communis*; MP+AS+SV+PC: mixture of Mongolian pine, *A*. *scoparia*, *S*. *viridis* and *P*. *communis*. Expected N mineralization amended by mixtures was calculated from the values amended by monocultures (S1B Fig) at 14, 42, 84 and 182 days of incubation according to Eq (1).
(DOCX)

S4 Fig Observed and expected soil respiration amended by litter mixtures at 14, 42, 84 and 182 days of incubation. Data are means ± SE, with *n* = 4. MP+AS: mixture of Mongolian pine + *A*. *scoparia*; MP+SV: mixture of Mongolian pine + *S*. *viridis*; MP+PC: mixture of Mongolian pine + *P*. *communis*; AS+SV: mixture of *A*. *scoparia* + *S*. *viridis*; AS+PC: mixture of *A*. *scoparia* + *P*. *communis*; SV+PC: mixture of *S*. *viridis* + *P*. *communis*; MP+AS+SV: mixture of Mongolian pine, *A*. *scoparia* and *S*. *viridis*; MP+AS+PC: mixture of Mongolian pine, *A*. *scoparia* and *P*. *communis*; MP+SV+PC: mixture of Mongolian pine, *S*. *viridis* and *P*. *communis*; AS+SV+PC: mixture of *A*. *scoparia*, *S*. *viridis* and *P*. *communis*; MP+AS+SV+PC: mixture of Mongolian pine, *A*. *scoparia*, *S*. *viridis* and *P*. *communis*. Expected respiration amended by mixtures was calculated from the values amended by monocultures (S1C Fig) at 14, 42, 84 and 182 days of incubation according to Eq (1).
(DOCX)

## Abbreviations

SpInt: species interaction
Day: incubation days
LG: lignin
CL: cellulose
SS: soluble sugar
PL: polyphenols
HP: hydrolyzable polyphenol
CT: condensed tannin
LG/N: lignin/N ratio
(LG+PL)/N: ratio of (lignin + polyphenol)/N
(LG+CL)/N: ratio of (lignin + cellulose)/N
MP: Mongolian pine
AS: *A*. *scoparia*
SV: *S*. *viridis*
PC: *P*. *communis*

## References

[1] Hättenschwiler S, Tiunov AV, Scheu S. Biodiversity and litter decomposition in terrestrial ecosystems. Annu Rev Ecol Evol S. 2005; 36:191–218. doi: 10.1146/annurev.ecolsys.36.112904.151932

[2] Hobbie SE. Plant species effects on nutrient cycling: revisiting litter feedbacks. Trends Ecol Evol. 2015; 20:1–7. doi: 10.1016/j.tree.2015.03.015 25900044 10.1016/j.tree.2015.03.015

[3] Townsend AR, Cleveland CC, Asner GP, Bustamante MMC. Controls over foliar N:P ratios in tropical rain forests. Ecology. 2007; 88:107–118. doi: 10.1890/0012-9658(2007)88[107:COFNRI]2.0.CO;2 17489459 10.1890/0012-9658(2007)88[107:cofnri]2.0.co;2

[4] Hättenschwiler S, Aeschlimann B, Coûteaux MM, Roy J, Bonal D. High variation in foliage and leaf litter chemistry among 45 tree species of a neotropical rainforest community. New Phytol. 2008; 179:165–175. doi: 10.1111/j.1469-8137.2008.02438.x 18422903 10.1111/j.1469-8137.2008.02438.x

[5] Rinnan R, Michelsen A, Jonasson S. Effects of litter addition and warming on soil carbon, nutrient pools and microbial communities in a subarctic heath ecosystem. Appl Soil Ecol. 2008: 39:271–281. doi: 10.1016/j.apsoil.2007.12.014

[6] McIntyre RES, Adams MA, Ford DJ, Grierson PF. Rewetting and litter addition influence mineralization and microbial communities in soils from a semi-arid intermittent stream. Soil Biol Biochem. 2009; 41:92–101. doi: 10.1016/j.soilbio.2008.09.021

[7] Wang QK, Wang SL, He TX, Liu L, Wu JB. Response of organic carbon mineralization and microbial community to leaf litter and nutrient additions in subtropical forest soils. Soil Biol Biochem. 2014; 71:13–20. doi: 10.1016/j.soilbio.2014.01.004

[8] Ma L, Guo C, Xin X, Yuan S, Wang R. Effects of belowground litter addition, increased precipitation and clipping on soil carbon and nitrogen mineralization in a temperate steppe. Biogeosciences. 2013; 10:7361–7372. doi: 10.5194/bg-10-7361-2013

[9] Hu YL, Wang SL, Zeng DH. Effects of single Chinese fir and mixed leaf litters on soil chemical, microbial properties and soil enzyme activities. Plant Soil. 2006; 282:379–386. doi: 10.1007/s11104-006-0004-5

[10] Aka H, Darici C. Carbon and nitrogen mineralization in carob soils with Kermes oak and Aleppo pine leaf litter. Eur J Soil Biol. 2005; 41:31–38. doi: 10.1016/j.ejsobi.2005.05.001

[11] Scheibe A, Gleixner G. Influence of litter diversity on dissolved organic matter release and soil carbon formation in a mixed beech forest. PLoS One. 2014; 9: e114040 doi: 10.1371/journal.pone.0114040 25486628 10.1371/journal.pone.0114040 PMC4259385

[12] Gartner TB, Cardon ZG. Decomposition dynamics in mixed-species leaf litter. Oikos. 2004; 104:230–246. doi: 10.1111/j.0030-1299.2004.12738.x

[13] Meier CL, Bowman WD. Chemical composition and diversity influence non-additive effects of litter mixures on soil carbon and nitrogen cycling: Implications for plant spcies loss. Soil Biol Biochem. 2010; 42:1447–1454. doi: 10.1016/j.soilbio.2010.05.005

[14] Jiang J, Li Y, Wang M, Zhou C, Cao G, Shi P, et al Litter species traits, but not richness, contribute to carbon and nitrogen dynamics in an alpine meadow on the Tibetan Plateau. Plant Soil. 2013; 373:931–941. doi: 10.1007/s11104-013-1859-x

[15] Gogo S, Laggoun-Défarge F, Merzouki F, Mounier S, Guirimand-Dufour A, Jozja N, Huguet A, et al In situ and laboratory non-additive litter mixture effect on C dynamics of *Sphagnum rubellum* and *Molinia caerulea* litters. J Soils Sediments. 2016; 16:13–27. doi: 10.1007/s11368-015-1178-3

[16] Loreau M, Naeem S, Inchausti P. Biodiversity and ecosystem functioning. Oxford University Press Oxford 2002.

[17] Chapin FS III, Zavaleta ES, Eviner VT, Naylor RL, Vitousek PM, Reynolds HL, et al Consequences of changing biodiversity. Nature. 2000; 405:234–242. doi: 10.1038/35012241 10821284 10.1038/35012241

[18] Kominoski J, Pringle C, Ball B, Bradford M, Coleman D, Hall D, et al Non-additive effects of leaf litter species diversity on breakdown dynamics in a detritus-based stream. Ecology. 2007; 88:1167–1176. doi: 10.1890/06-0674 17536403 10.1890/06-0674

[19] Duan J, Wang S, Zhang Z, Xu G, Luo C, Chang X, et al Non-additive effect of species diversity and temperature sensitivity of mixed litter decomposition in the alpine meadow on Tibetan Plateau. Soil Biol Biochem. 2013; 57:841–847. doi: 10.1016/j.soilbio.2012.08.009

[20] Meier CL, Bowman WD. Links between plant litter chemistry, species diversity, and below-ground ecosystem function. PNAS. 2008; 105:19780–19785. doi: 10.1073/pnas.0805600105 19064910 10.1073/pnas.0805600105 PMC2604978

[21] Cong WF, Ruijven J, Mommer L, De Deyn GB, Berendse F, Hoffland E. Plant species richness promotes soil carbon and nitrogen stocks in grasslands without legumes. J Ecol. 2014; 102:1163–1170. doi: 10.1111/1365-2745.12280

[22] Chen Y, Sun J, Xie F, Wang X, Cheng G, Lu X. Litter chemical structure is more important than species richness in affecting soil carbon and nitrogen dynamics including gas emissions from an alpine soil. Biol Fertil Soils. 2015; 51:791–800. doi: 10.1007/s00374-015-1025-0

[23] Ball BA, Carrillo Y, Molina M. The influence of litter composition across the litter–soil interface on mass loss, nitrogen dynamics and the decomposer community. Soil Biol Biochem. 2014; 69:71–82. doi: 10.1016/j.soilbio.2013.10.048

[24] Hättenschwiler S, Vitousek PM. The role of polyphenols in terrestrial ecosystem nutrient cycling. Trends Ecol Evol. 2000 15:238–243. doi: 10.1016/S0169-5347(00)01861-9 10802549 10.1016/s0169-5347(00)01861-9

[25] Smolander A, Kanerva S, Adamczyk B, Kitunen V. Nitrogen transformations in boreal forest soils—does composition of plant secondary compounds give any explanations? Plant Soil. 2012; 350:1–26. doi: 10.1007/s11104-011-0895-7.

[26] Kraus TEC, Zasoski RJ, Dahlgren RA, Horwath WR, Preston CM. Carbon and nitrogen dynamics in a forest soil amended with purified tannins from different plant species. Soil Biol Biochem. 2004; 36:309–321. doi: 10.1016/j.soilbio.2003.10.006

[27] Kraus TEC, Dahlgren RA, Zasoski RJ. Tannins in nutrient dynamics of forest ecosystems: A review. Plant Soil. 2003; 256:41–66. doi: 10.1023/A:1026206511084

[28] Boerjan W, Ralph J, Baucher M. Lignin biosynthesis. Annu Rev Plant Biol. 2003; 54:519–546. doi: 10.1146/annurev.arplant.54.031902.134938 14503002 10.1146/annurev.arplant.54.031902.134938

[29] Talbot JM, Treseder KK. Interactions among lignin, cellouse, and nitrogen drive litter chemistry-decay relationships. Ecology. 2012; 93:345–354. doi: 10.1890/11-0843.1 22624316 10.1890/11-0843.1

[30] Hoorens B, Aerts R, Stroetenga M. Does initial litter chemistry explain litter mixture effects on decomposition? Oecologia. 2003; 137:578–586. doi: 10.1007/s00442-003-1365-6 14505026 10.1007/s00442-003-1365-6

[31] Lummer D, Scheu S, Butenschoen O. Connecting litter quality, microbial community and nitrogen transfer mechanisms in decomposing litter mixtures. Oikos. 2012; 121:1649–1655. doi: 10.1111/j.1600-0706.2011.20073.x

[32] Redin M, Recous S, Aita C, Dietrich G, Skolaude AC, Ludke WH, et al How the chemical composition and heterogeneity of crop residue mixtures decomposing at the soil surface affects C and N mineralization. Soil Biol Biochem. 2014; 78:65–75. doi: 10.1016/j.soilbio.2014.07.014

[33] Petchey OL, Gaston KJ. Functional diversity: Back to basics and looking forward. Ecol Lett. 2006; 9:741–758. doi: 10.1111/j.1461-0248.2006.00924.x 16706917 10.1111/j.1461-0248.2006.00924.x

[34] Rudolf VHW, Rasmussen NL. Ontogenetic functional diversity: Size structure of a keystone predator drives functioning of a complex ecosystem. Ecology. 2013; 94:1046–1056. doi: 10.1890/12-0378.1 23858645 10.1890/12-0378.1

[35] Naeem S, Wright JP. Disentangling biodiversity effects on ecosystem functioning: deriving solutions to a seemingly insurmountable problem. Ecol Lett. 2003; 6:567–579. doi: 10.1046/j.1461-0248.2003.00471.x

[36] Spehn EM, Hector A, Joshi J, Scherer-Lorenzen M, Schmid B, Bazeley-White E, et al Ecosystem effects of biodiversity manipulations in European grasslands. Ecolo Mono. 2005; 75:37–63. doi: 10.1890/03-4101

[37] Villéger S, Mason NW, Mouillot D. New multidimensional functional diversity indices for a multifaceted framework in functional ecology. Ecology. 2008; 89: 2290–2301. doi: 10.1890/07-1206.1 18724739 10.1890/07-1206.1

[38] Mao B, Yu ZY, Zeng DH. Non-additive effects of species mixing on litter mass loss and chemical properties in a Mongolian pine plantation of Northeast China. Plant Soil. 2015; 396:339–351. doi: 10.1007/s11104-015-2593-3

[39] Zeng DH, Hu YL, Chang SX, Fan ZP. Land cover change effects on soil chemical and biological properties after planting Mongolian pine (*Pinus sylvestris* var. *mongolica*) in sandy lands in Keerqin, northeastern China. Plant Soil. 2009; 317:121–133. doi: 10.1007/s11104-008-9793-z

[40] Mulvaney RL. Nitrogen—Inorganic forms In: Sparks DL, Page AL, Helmke PA, Loeppert RH, Soltanpour PN, Tabatabai MA, Johnston CT, Sumner ME, editors. Methods of Soil Analysis, Part 3: Chemical Methods. Madison: Soil Science Society of America, American Society of Agronomy 1996 pp. 1123–1184.

[41] Brookes PC, Landman A, Pruden G, Jenkinson DS. Chloroform fumigation and the release of soil nitrogen: a rapid direct extraction method for measuring microbial biomass nitrogen in soil. Soil Biol Biochem. 1985; 17:837–842. doi: 10.1016/0038-0717(85)90144-0

[42] Cabrera ML, Beare MH. Alkaline persulfate oxidation for determining total nitrogen in microbial biomass extracts. Soil Sci Soc Am J. 1993; 57:1007–1012. doi: 10.2136/sssaj1993.03615995005700040021x

[43] Nelson DW, Sommers LE. Total carbon, organic carbon and organic matter, In: Sparks DL, Page AL, Helmke PA, Loeppert RH, Soltanpour PN, Tabatabai MA, Johnston CT, Sumner ME, editors. Methods of Soil Analysis, Part 3: Chemical Methods. Madison: Soil Science Society of America 1996 pp. 961–1010.

[44] Iiyama K, Wallis AFA, Determination of lignin in herbaceous plants by an improved acetyl bromide procedure. J Sci Food Agr. 1990; 51:145–161. doi: 10.1002/jsfa.2740510202

[45] Updegraff DM. Semimicro determination of cellulose in biological materials. Anal Biochem. 1969; 32:420–424. doi: 10.1016/S0003-2697(69)80009-6 5361396 10.1016/s0003-2697(69)80009-6

[46] Helbert JR, Brown KD. Color reaction of anthrone with monosaccharide mixtures and oligo- and polysaccharides containing hexuronic acids. Anal Chem. 1957; 29:1464–1466. doi: 10.1021/ac60130a020

[47] Waterman PG, Mole S. Analysis of Phenolic Plant Metabolites, The Methods in Ecology Series. Blackwell Scientific Publications Oxford 1994.

[48] Porter LJ, Hrstich LN, Chan BG. The conversion of procyanidins and prodelphinidins to cyanidin and delphinidin. Phytochemistry. 1986; 25:223–230. doi: 10.1016/S0031-9422(00)94533-3

[49] Ball BA, Hunter MD, Kominoski JS, Swan CM, Bradford MA. Consequences of non-random species loss for decomposition dynamics: experimental evidence for additive and non-additive effects. J Ecol. 2008; 96:303–313. doi: 10.1111/j.1365-2745.2007.01346.x

[50] Salamanca EF, Kaneko N, Katagiri S. Effects of leaf litter mixtures on the decomposition of *Quercus serrata* and *Pinus densiflora* using field and laboratory microcosm methods. Ecol Eng. 1998; 10:53–73. doi: 10.1016/S0925-8574(97)10020-9

[51] Wardle DA, Yeates GW, Barker GM, Bonner KI. The influence of plant litter diversity on decomposer abundance and diversity. Soil Biol Biochem. 2006; 38:1052–1062. doi: 10.1016/j.soilbio.2005.09.003

[52] Li W, Pan K, Wu N, Wang J, Han C, Liang X. Effects of mixing pine and broadleaved tree/shrub litter on decomposition and N dynamics in laboratory microcosms. Ecol Res. 2009; 24:761–769. doi: 10.1007/s11284-008-0546-5

[53] Bottollier-Curtet M Charcosset JY, Planty-Tabacchi AM, Tabacchi E. Chemical composition rather than plant geographic origin drives the breakdown of riparian plant litter with changes in associated invertebrate diversity. Plant Soil. 2015; 390:265–278. doi: 10.1007/s11104-015-2394-8

[54] Sayer EJ. Using experimental manipulation to assess the roles of leaf litter in the functioning of forest ecosystems. Biol Rev. 2006; 81:1–31. doi: 10.1017/S1464793105006846 16460580 10.1017/S1464793105006846

[55] Han T, Huang W, Liu J, Zhou G, Xiao Y. Different soil respiration responses to litter manipulation in three subtropical successional forests. Sci Rep. 2015; 5:18166 doi: 10.1038/srep18166 26656136 10.1038/srep18166 PMC4676067

[56] Leff JW, Wieder WR, Taylor PG, Townsend AR, Nemergut DR, Grandy AS, et al Experimental litterfall manipulation drives large and rapid changes in soil carbon cycling in a wet tropical forest. Glob Chang Biol. 2012; 18:2969–2979. doi: 10.1111/j.1365-2486.2012.02749.x 24501071 10.1111/j.1365-2486.2012.02749.x

[57] Fang X, Zhao L, Zhou G, Huang W, Liu J. Increased litter input increases litter decomposition and soil respiration but has minor effects on soil organic carbon in subtropical forests. Plant Soil. 2015; 392:139–153. doi: 10.1007/s11104-015-2450-4

[58] Wardle DA, Nilsson MC, Zackrisson O, Gallet C. Determinants of litter mixing effects in a Swedish boreal forest. Soil Biol Biochem. 2003; 35:827–835. doi: 10.1016/S0038-0717(03)00118-4

[59] Gießelmann UC, Martins KG, Brändle M, Schädler M, Marques R, Brandl R. Diversity and ecosystem functioning: litter decomposition dynamics in the Atlantic rainforest. Appl Soil Ecol. 2010; 46:283–290. doi: 10.1016/j.apsoil.2010.07.006

[60] Schädler M, Brandl R. Do invertebrate decomposers affect the disappearance rate of litter mixtures? Soil Biol Biochem. 2005; 37:329–337. doi: 10.1016/j.soilbio.2004.07.042

[61] Nilsson MC, Wardle DA. Understory vegetation as a forest ecosystem driver: evidence from the northern Swedish boreal forest. Fron Ecol Environ. 2005; 3:421–428. doi: 10.1890/1540-9295(2005)003[0421:UVAAFE]2.0.CO;2

[62] Xu S, Liu LL, Sayer EJ. Variability of above-ground litter inputs alters soil physicochemical and biological processes: a meta-analysis of litterfall-manipulation experiments. Biogeosciences. 2013; 10:7423–7433. doi: 10.5194/bg-10-7423-2013

[63] Muller RN. Nutrient relations of the herbaceous layer in deciduous forest ecosystem In: Gilliam FS, Roberts MR, editors. The Herbaceous Layer in Forests of Eastern North America. New York: Oxford University Press 2003 pp. 15–37.

