# Supplementary material for: Species diversity and chemical properties of litter influence non-additive effects of litter mixtures on soil carbon and nitrogen cycling
Source: PLoS One. 2017 Jul 7;12(7):e0180422. doi: 10.1371/journal.pone.0180422 (PMC5501526; doi:10.1371/journal.pone.0180422)
Supplement: S1 Table — (DOCX) [file pone.0180422.s001.docx]

**S1 Table** Species diversity (*H*c), species richness and species composition of single litter species and their mixtures

|  | *H*c | Species richness | Species composition | SpInt |
| --- | --- | --- | --- | --- |
| Monoculture |  |  |  |  |
| MP |  | 1 | 1 |  |
| AS |  | 1 | 2 |  |
| SV |  | 1 | 3 |  |
| PC |  | 1 | 4 |  |
| Mixture |  |  |  |  |
| MP+AS | 1.24 | 2 | 5 | 1 |
| MP+SV | 1.24 | 2 | 6 | 2 |
| MP+PC | 1.22 | 2 | 7 | 3 |
| AS+SV | 1.21 | 2 | 8 | 4 |
| AS+PC | 1.16 | 2 | 9 | 5 |
| SV+PC | 1.16 | 2 | 10 | 6 |
| MP+AS+SV | 1.15 | 3 | 11 | 7 |
| MP+AS+PC | 1.2 | 3 | 12 | 8 |
| MP+SV+PC | 1.2 | 3 | 13 | 9 |
| AS+SV+PC | 1.16 | 3 | 14 | 10 |
| MP+AS+SV+PC | 1.2 | 4 | 15 | 11 |

Species diversity (*H*c) was calculated from the values of the initial concentration of C, N, lignin, Cellulose, soluble sugar, polyphenol, condensed tannin, hydrolyzable polyphenol of four species litter according to Eq. (4).

SpInt is abbreviated as species interaction

MP: Mongolian pine; AS: *A.* *scoparia*; SV: *S. viridis*; PC: *P. communis*; MP+AS: mixture of Mongolian pine + *A.* *scoparia*; MP+SV: mixture of Mongolian pine + *S. viridis*; MP+PC: mixture of Mongolian pine + *P. communis*; AS+SV: mixture of *A.* *scoparia* + *S. viridis*; AS+PC: mixture of *A.* *scoparia* + *P. communis*; SV+PC: mixture of *S. viridis* + *P. communis*; MP+AS+SV: mixture of Mongolian pine, *A.* *scoparia* and *S. viridis*; MP+AS+PC: mixture of Mongolian pine, *A.* *scoparia* and *P. communis*; MP+SV+PC: mixture of Mongolian pine, *S. viridis* and *P. communis*; AS+SV+PC: mixture of *A.* *scoparia*, *S. viridis* and *P. communis*; MP+AS+SV+PC: mixture of Mongolian pine, *A.* *scoparia*, *S. viridis* and *P. communis*.
