# Supplementary material for: Species diversity and chemical properties of litter influence non-additive effects of litter mixtures on soil carbon and nitrogen cycling
Source: PLoS One. 2017 Jul 7;12(7):e0180422. doi: 10.1371/journal.pone.0180422 (PMC5501526; doi:10.1371/journal.pone.0180422)
Supplement: S3 Fig — Data are means ± SE, with n = 4. MP+AS: mixture of Mongolian pine + A. scoparia; MP+SV: mixture of Mongolian pine + S. viridis; MP+PC: mixture of Mongolian pine + P. communis; AS+SV: mixture of A. scoparia + S. viridis; AS+PC: mixture of A. scoparia + P. communis; SV+PC: mixture of S. viridis + P. communis; MP+AS+SV: mixture of Mongolian pine, A. scoparia and S. viridis; MP+AS+PC: mixture of Mongolian pine, A. scoparia and P. communis; MP+SV+PC: mixture of Mongolian pine, S. viridis and P. communis; AS+SV+PC: mixture of A. scoparia, S. viridis and P. communis; MP+AS+SV+PC: mixture of Mongolian pine, A. scoparia, S. viridis and P. communis. Expected N mineralization amended by mixtures was calculated from the values amended by monocultures (S1B Fig) at 14, 42, 84 and 182 days of incubation according to Eq (1). (DOCX) [file pone.0180422.s004.docx]

**S3 Fig.**
